# Hypophosphatemia in suspected seizures evaluated in first seizure clinics and neurology consults

**DOI:** 10.1002/epi4.70172

**Published:** 2025-10-27

**Authors:** Sophie N. M. Binks, Daniil Zorkin, Bernard Liem, Arjune Sen

**Affiliations:** ^1^ Department of Neurology John Radcliffe Hospital, Oxford University Hospitals NHS Foundation Trust Oxford UK; ^2^ Oxford Epilepsy Research Group, Nuffield Department of Clinical Neurosciences University of Oxford Oxford UK

**Keywords:** biomarker, electrolyte, epilepsy, hypophosphatemia, seizure, telemedicine

## Abstract

Transient loss of consciousness (TLoC) is a leading cause of referrals to acute neurological services. A witness account is often lacking, and ancillary investigations are a critical diagnostic adjunct. Hypophosphatemia was recently identified as a potential marker of epileptic seizures in ward and emergency department presentations. We evaluated the real‐world utility of checking phosphate in an unselected cohort of people presenting with TLoC. We retrospectively reviewed 182 episodes (91 referrals to the first seizure clinic and 91 consults) from 170 patients. We assessed nine prespecified serological markers frequently measured in people presenting with TLoC. Raw *p*‐values comparing mean levels showed a significant difference between epileptic seizures and other causes of TLoC only for phosphate (0.98 vs 1.17 mmol/L, *p* = 0.01) and lactate (2.93 vs 1.77 mmol/L, *p* = 0.001). Mean lactate level, but not phosphate, remained significant after multiple comparison correction (*p* = 0.01). However, a phosphate below 0.8 mmol/L was significantly more likely to be associated with epileptic seizures (17/60; 28%) than episodes not thought to be epileptic (2/30; 6.6%; *p* = 0.03). Stepwise regression showed a model including lactate, phosphate, and calcium predicted convulsive seizures with an area under the ROC curve of 0.825 (95% CI 0.718–0.931), superseding lactate alone. Checking serum phosphate may be valuable in helping to determine the etiology of an episode of TLoC.

**Plain Language Summary:**

We studied blood test results of 170 people suspected of having had an epileptic seizure who presented to a UK hospital neurology service. Although blood phosphate tests were infrequently requested, our results suggest a low phosphate level could be useful to help distinguish between epileptic seizures and other causes of collapse.

**Key points**

- Serum phosphate measurements are rarely included in the acute evaluation of Transient Loss of Consciousness (TLoC).
- Measured within 6 h of an ictal episode, low serum phosphate can aid in identification of an epileptic seizure.
- Absolute levels below the institutional lower limit of normal were significantly associated with epileptic activity.
- Combination with serum lactate and calcium identified convulsive seizure with an AUC of 0.825 (0.718–0.931).
- Our findings support prior studies that serum phosphate may be a valuable supportive test in TLoC work‐up.

## 1 INTRODUCTION

Transient loss of consciousness (TLoC) events, incorporating both suspected seizures and other causes of blackouts, are a leading cause of acute neurological presentations. They account for ~1% of Emergency Department (ED) visits^1^ and around one sixth of new referrals to Neurology Outpatient Departments.^2^ In most institutions, daily neurological practice involves multiple assessments for people presenting with TLoC, including phone consults and telehealth. In these circumstances, precise seizure classification may not be possible, although advice on episode management is still required. A witness account is essential in differentiating between different causes of TLoC, yet in the UK‐based National Audit of Seizure Management in Hospitals, this was only attempted in approximately 70% of first seizure evaluations at the point of contact.^3^ Ancillary evidence provided by supporting investigations can, therefore, be a useful diagnostic adjunct.

Phosphate was recently shown to be the most profoundly altered electrolyte after generalized tonic–clonic seizures.^4^ Hypophosphatemia has since emerged as a potential biomarker of epileptic seizures compared to other causes of TLoC in European EDs.^5^, ^6^, ^7^ Low phosphate has also been detected in other mammalian species, for example dogs presenting with seizures.^8^ The frequency with which phosphate levels are checked for people presenting with TLoC in routine clinical care remains to be clarified.

We audited the frequency with which phosphate was measured in TLoC presentations in a real‐world UK setting. Our goal was to understand the utility of post‐ictal phosphate levels in accurately differentiating epileptic seizures from non‐epileptic episodes, including functional dissociative seizures (FDS), in undifferentiated patients.

## 2 METHODS

We retrospectively identified cases that were referred to either a first seizure clinic (2021–2023) or to hospital‐based on‐call neurological services (February–June 2023). Clinical variables captured were age, sex, and time to blood test. Recorded serological variables consisted of nine prespecified markers in: hematology (neutrophil count); electrolytes (sodium, potassium, calcium, magnesium, and phosphate); infection (C‐reactive protein [CRP]); and metabolism (glucose and lactate).

Each episode was rated as an epileptic seizure or not epileptic (including FDS) through detailed notes review. Where a final disposition was not reached, three authors (SNB, AS, and BL) reviewed the entire case file to best determine whether the event was epileptic in etiology or not. Cases were also reviewed by SNB, AS, and BL against International League Against Epilepsy (ILAE) classifications.^9^ This was done independent of serological findings.

Statistics were performed in R (v4.0.3 and v4.4.0). We compared between‐group differences for demographic and biochemical features in the epileptic seizure and non‐epileptic groups with binomial tests (chi‐squared or Fisher's exact test for categorical variables, *t*‐test or Wilcoxon test for continuous variables). Fisher's test was used if any group compared five or fewer individuals. *t*‐test was used for normally, and Wilcoxon test for non‐normally, distributed data, with normality assessed via the Shapiro–Wilk test. Correction for multiple comparisons was performed with Holm's method. Parameters collected via blood gas machine or laboratory were considered concurrent if results were timestamped within a 1‐h slot. We used Spearman's test for correlation. Logistic regression was carried out in base R, stepwise regression using the MASS function, and AUC with the pROC function. Data visualization was handled with ggplot2 and pROC. Missing data were excluded from relevant analyses. Significance was set at two‐sided *p* < 0.05.

The audit was registered with Oxford University Hospitals NHS Foundation Trust; reference number 8576.

## 3 RESULTS

**FIGURE 1:**
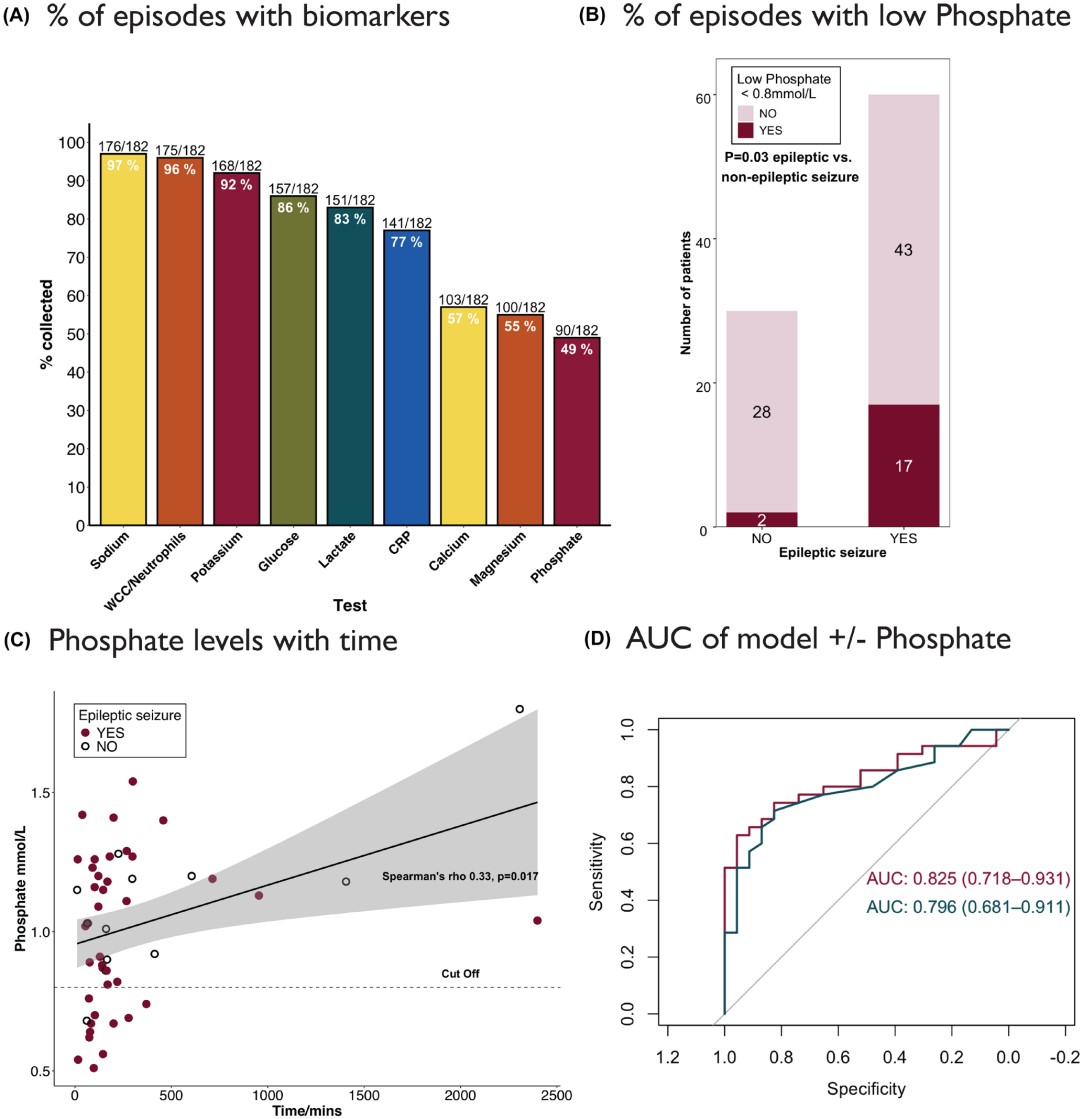
(A) Bar chart showing proportion of episodes in which the prespecified variables were collected, plotted in descending order of frequency. Numbers at the top of the bar give proportion of episodes in which each parameter was collected, and those within the bar represent the percentage. (B) Stacked bar chart. A phosphate <0.8 mmol/L was present in 28% (17/60) epileptic seizures and 6.6% (2/30) non‐epileptic episodes (*p* = 0.03) (Fisher's exact test; dark red shows low phosphate and pale red shows phosphate in normal range). (C) Scatterplot of phosphate levels (*y* axis) against time from episode onset (*x* axis). Values below the threshold (<0.8 mmol/L) were only detected at a maximum of 6.10 h. Dark red dots represent seizure cases and open black dots, non‐epileptic seizure cases. The dotted line marks the cutoff for a low phosphate level in our institution (<0.8 mmol/L). Shaded area = standard error. (D) AUC of a logistic regression model including only lactate level (blue line) or the result of a stepwise regression including lactate, phosphate and calcium (dark red line). AUC values with 95% confidence intervals are depicted on the graph. AUC, area under the curve; CRP, C‐reactive protein; WCC, white cell count.

Altogether, 182 episodes (91 each first seizure clinic/liaison consultation) were analyzed. Overall, 120 (66%) were epileptic seizures. These events derived from 170 patients (77 women, mean age 51.5 years). Full demographic details and outcome diagnoses are summarized in Table 1. The proportion of requested blood tests across the nine prespecified parameters ranged from 176/182 (97%) for sodium to 90/182 (49%) for phosphate (Figure 1A). Timestamps were consistent with blood gas and laboratory tests being part of the same blood draw for all but 10 cases.

**TABLE 1:** (A) Key demographics by total cohort, and by patients referred to first seizure clinic or consults service. (B) Diagnoses of epilepsy (120) or non‐epileptic (62) episodes.

	All (*n* = 170)	1st seizure clinic (*n* = 85)	Consults (*n* = 85)	Raw *p* first seizure versus consults	Corrected *p* ^d^
*(A) Key demographics*					
Age in years mean (median, range)	51.5 (51, 16–92)	48 (49, 16–88)	54 (54, 16–92)	0.06	0.27
Sex female (*n*, %)	77/170 (45%)	41/85 (48%)	36/85 (42%)	0.54	1
Median time to blood tests/hours per episode (range)	2.45 (11 min to 55.10 h)^a^	2.41 (16 min to 55.10 h)^b^	2.46 (11 min to 39.59 h)^c^	0.48	1
% epileptic seizure episodes (*n*, %)	120/182 (66%)	63/91 (69%)	57/91 (63%)	0.44	1
*(B) Diagnostic outcomes*					
Epilepsy (120 episodes)	Convulsive seizure (56), Epileptic encephalopathy (2), Focal seizure (4), Focal aware seizure (8), Focal to bilateral tonic‐clonic seizure (20), Focal unaware seizure (22), Other generalized seizure (3), Seizure—Unknown Type (3), and Status Epilepticus (2)				
Non‐epileptic (62 episodes)	Amyloid (2), Cardiac (2), Catatonia (1), Cerebrovascular (2), Delirium (2), Encephalopathy (1), FDS (14), Hypophysitis (1), Hypotension (2), Intoxication (1), Migraine (2), Migraine + FDS (1), Parkinson's Disease (1), Prolonged post ictal (1), PPPD (2), Psychosis (1), Sepsis (1), Post‐surgical palsy (1), Syncope (14), TGA (2), and TLoC—Unknown Cause (8)				

Abbreviations: FDS, functional dissociative seizures; *n*, number; PPPD, Persistent Postural‐Perceptual Dizziness; TGA, Transient Global Amnesia; TLoC, Transient Loss of Consciousness.

^a^ Available for 92 episodes.

^b^ Available for 43 episodes.

^c^ Available for 49 episodes.

^d^ Holm corrected.

Per‐group biochemical parameters are shown in Table 2. Raw *p*‐values comparing mean levels showed a significant difference between epileptic seizure and non‐epileptic events only for phosphate (0.98 vs 1.17 mmol/L; *p* = 0.01) and lactate (2.93 vs 1.77 mmol/L; *p* = 0.001). Only lactate levels remained significant after multiple comparison correction (*p* = 0.01). However, a phosphate below the lower limit of normal in our institution (<0.8 mmol/L) was significantly more likely in an epileptic seizure than in episodes that were not epileptic (17/60, 28% vs 2/30, 6.6%, *p* = 0.03) (Figure 1B). The seizure type in individuals with low phosphate levels was convulsive in nine, focal to bilateral tonic‐clonic in five, focal in two, and status epilepticus in one (Table S1). Of 15 people with FDS, one had a low phosphate level—this individual having a concomitant diagnosis of epilepsy, although the index event was diagnosed as FDS. The other low phosphate result in the non‐epileptic group was associated with metabolic encephalopathy.

**TABLE 2:** Per‐group biochemical parameters.

Parameter—mean (median, range)	All (*n* = 182 episodes)	Epileptic seizure (*n* = 120 episodes)	Non‐epileptic (*n* = 62 episodes)	Raw *p* epileptic seizure versus non‐epileptic episode	Corrected *p* ^a^
Sodium (mmol/L)	138 (139, 121–147)	138 (139, 121–147)	138 (139, 126–143)	0.87	1
Neutrophils (×10^9^/L)	7.41 (6.5, 1.32–20.82)	7.40 (6.52, 1.65–19.64)	7.43 (6.16, 1.32–20.82)	0.66	1
Potassium (mmol/L)	4.10 (4.10, 2.9–5.5)	4.04 (4, 2.9–5.2)	4.18 (4.2, 3.3–5.5)	0.05	0.38
Glucose (mmol/L)	6.66 (6, 3.3–15.7)	6.63 (6, 3.8–15.7)	6.73 (6.2, 3.3–14.3)	0.74	1
Lactate (mmol/L)	2.55 (1.5, 0.6–19)	2.93 (1.8, 0.6–19)	1.77 (1.2, 0.6–11.9)	0.001	0.01
CRP (mg/L)	15.38 (3.4, 0.2–236.2)	10.9 (3.1, 0.2–91.6)	24.1 (3.75, 0.3–236.2)	0.30	1
Calcium (mmol/L)	2.35 (2.33, 2.15–2.60)	2.34 (2.32, 2.15–2.60)	2.37 (2.36, 2.19–2.60)	0.05	0.38
Magnesium (mmol/L)	0.82 (0.82, 0.57–1.16)	0.82 (0.82, 0.57–1.16)	0.82 (0.82, 0.65–0.94)	0.98	1
Phosphate (mmol/L)	1.04 (1.08, 0.42–2.13)	0.98 (0.98, 0.42–1.54)	1.17 (1.16, 0.66–2.13)	0.01	0.09

^a^ Holm corrected.

There was a modest, but significant, correlation between phosphate level and time from blood draw. All phosphate levels below the threshold for the lower limit of normal were taken within 6.10 h from episode onset (Figure 1C). Taken together, these findings are consistent with a post‐ictal decrease in phosphate being detectable at up to 6 h post‐ictally. There was also a significant negative correlation between lactate levels and time from blood draw (Figure S1).

Finally, we examined the predictive value of phosphate in differentiating epileptic seizures from events that were not epileptic. We evaluated levels of all entities attaining ≤*p* = 0.05 between the groups (potassium, calcium, phosphate, and lactate levels) via a stepwise regression model. The best fit model for any seizure included only lactate, with an AUC of 0.771 (95% CI 0.659–0.884), borderline superior to a model with lactate and phosphate (AUC 0.768 [95% CI 0.656–0.880]) (Figure S2). When considering only convulsive seizures, a combined stepwise model including phosphate, calcium, and lactate exhibited a better AUC of 0.825 (95% CI 0.718–0.931) compared to lactate alone (0.796, 95% CI 0.681–0.911; Figure 1D and Supporting Information). A stand‐alone logistic regression model with an absolute low phosphate was significant (*p* = 0.01) with an odds ratio of 5.5 in predicting any epileptic seizure, although confidence intervals were wide (95% CI 1.19–25.83) (Supporting Information).

## 4 DISCUSSION

We examined the frequency with which phosphate is incorporated into the work‐up of TLoC in a real‐world setting and its role in differentiating epileptic seizures from non‐epileptic events. Our investigation covered both in‐person and remote reviews to better reflect what occurs in acute neurological services.

Despite growing evidence that it is the most altered electrolyte in post‐seizure physiology, phosphate was also the most rarely requested in this cohort. The low proportion of episodes in which phosphate was requested in routine practice highlights the potential to improve awareness of its potential utility. It is likely the non‐significant adjusted *p*‐values for numeric phosphate levels in epileptic seizure compared to non‐epileptic events, and wide confidence intervals in a stand‐alone logistic regression model, reflect, at least in part, loss of power due to missing data. An absolute phosphate level below 0.8 mmol/L (the lower limit of normal at our institution) was more likely in epileptic compared to non‐epileptic episodes. Checking phosphate levels therefore represents a simple, inexpensive, and helpful addition to the assessment of TLoC events.

Our results, in keeping with former studies, also suggest that checking phosphate levels may be informative up to 6 h after ictal onset.^4^, ^5^ The mechanism of hypophosphatemia is not confirmed but potentially includes hormonal or exertional effects.^5^, ^10^ Lactate levels also diminished over time, reinforcing a message that timely investigations are essential to maximize deductive information in suspected seizure disorders.

The AUC of lactate alone was comparable to lactate combined with phosphate to detect any seizure, but a stepwise model also including phosphate and calcium did modestly increase the ability to discriminate convulsive epileptic seizures from other events. This could be of particular value when a witness account is not available or in non‐specialist settings. Testing of phosphate in low to middle‐income countries, especially if done soon after an event, may enable primary healthcare workers to better determine the potential cause of an episode of TLoC, streamlining initial decision‐making and facilitating appropriate onward referral. That there was only one low phosphate in the 15 FDS episodes may also point to a role in distinguishing epileptic seizures from functional attacks.

Our study is consistent with previous work linking hypophosphatemia to generalized convulsive tonic–clonic seizures.^4^, ^5^, ^6^, ^8^ We only found low phosphate levels in two people with focal seizures without generalization. Hypophosphatemia has recently been reported in connection with autoimmune encephalitis with antibodies to leucine‐rich glioma‐inactivated 1 encephalitis, a condition that usually presents with frequent focal seizures.^11^ Larger studies will, though, be required to determine whether low phosphate is also a biomarker in focal seizure types.

## 5 LIMITATIONS

As with any real‐world study, there are limitations owing to missing data and that review of medical records could not always confirm the type of seizure. The diagnosis of a seizure, except where concurrent EEG captures an event, remains a clinical diagnosis and should not be ruled in or out by a single biomarker. Our number of FDS episodes was low, and future work should recruit more individuals with FDS to better understand the role of phosphate in this cohort. Lactate levels can be affected by pre‐analytical storage and patient factors, which cannot be ruled out in busy emergency departments and/or medical wards.^12^ Because phosphate levels between epileptic and non‐epileptic events were not significant after multiple comparison correction, it is possible this could represent a false positive finding.

Overall, our study adds to the evidence that serum phosphate can be a helpful supportive test in evaluating potential seizure episodes. We would propose adding serum phosphate to ED first seizure caresets as part of a battery of tests requested when someone presents with TLOC. Prospective studies should audit the utility of such incorporation in improving diagnostic accuracy.

## CONFLICT OF INTEREST STATEMENT

Dr. Binks, Dr. Zorkin, Dr. Liem, and Professor Sen have no relevant conflicts of interest.

## ETHICAL PUBLICATION STATEMENT

We confirm that we have read the journal's position on issues involved in ethical publication and affirm that this report is consistent with those guidelines.

## PATIENT CONSENT STATEMENT

The audit was registered with Oxford University Hospitals NHS Foundation Trust; reference number 8576.

## Supporting information


**Supplementary Figure 1.** Scatterplot of lactate levels (y axis) against time from episode onset (x axis). Dark red dots represent seizure cases and open black dots, non‐epileptic seizure cases. The dotted line marks zero, as there is no formal cutoff level for lactate. Lactate was collected at the same time as laboratory bloods in all but 10 cases, of which four had a documented episode time and are included here. Shaded area = standard error.
**Supplementary Figure 2.** AUC of a logistic regression model including only lactate level (blue line) or lactate with phosphate level (dark red line) to detect any seizure. AUC values with 95% confidence intervals are depicted on the graph. AUC, area under the curve.
**Supplementary Table S1.** Absolute low phosphate levels detected in seizure types with or without a convulsive component. Fisher’s exact test to compare the two groups was non‐significant (*p* = 0.07).

## Data Availability

The data that support the findings of this study are available on request from the corresponding author. The data are not publicly available due to privacy or ethical restrictions.
