# Supplementary material for: Hypophosphatemia in suspected seizures evaluated in first seizure clinics and neurology consults
Source: Epilepsia Open. 2025 Oct 27;10(6):2025–31. doi: 10.1002/epi4.70172 (PMC12716309; doi:10.1002/epi4.70172)
Supplement: Supplementary file 1 — Supplementary Figure 1. Scatterplot of lactate levels (y axis) against time from episode onset (x axis). Dark red dots represent seizure cases and open black dots, non‐epileptic seizure cases. The dotted line marks zero, as there is no formal cutoff level for lactate. Lactate was collected at the same time as laboratory bloods in all but 10 cases, of which four had a documented episode time and are included here. Shaded area = standard error. Supplementary Figure 2. AUC of a logistic regression model including only lactate level (blue line) or lactate with phosphate level (dark red line) to detect any seizure. AUC values with 95% confidence intervals are depicted on the graph. AUC, area under the curve. Supplementary Table S1. Absolute low phosphate levels detected in seizure types with or without a convulsive component. Fisher’s exact test to compare the two groups was non‐significant (p = 0.07). [file EPI4-10-2025-s001.docx]

**Supplementary Figures**

**
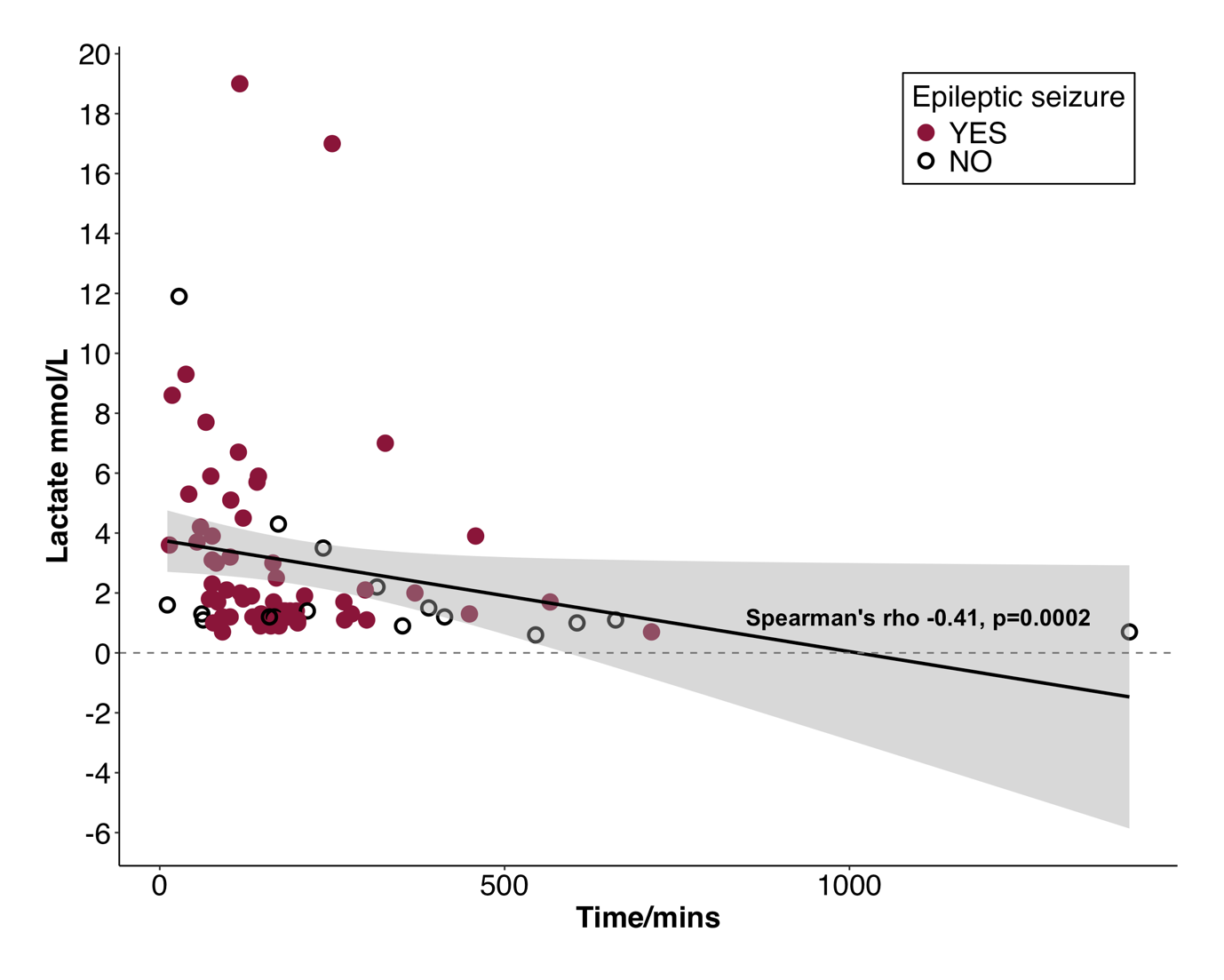
**

**Supplementary Figure 1.** Scatter plot of lactate levels (y axis) against time from episode onset (x axis). Dark red dots represent seizure cases and open black dots, non-epileptic seizure cases. The dotted line marks zero, as there is no formal cut-off level for lactate. Lactate was collected at the same time as laboratory bloods in all but 10 cases, of which four had a documented episode time and are included here. Shaded area = standard error.


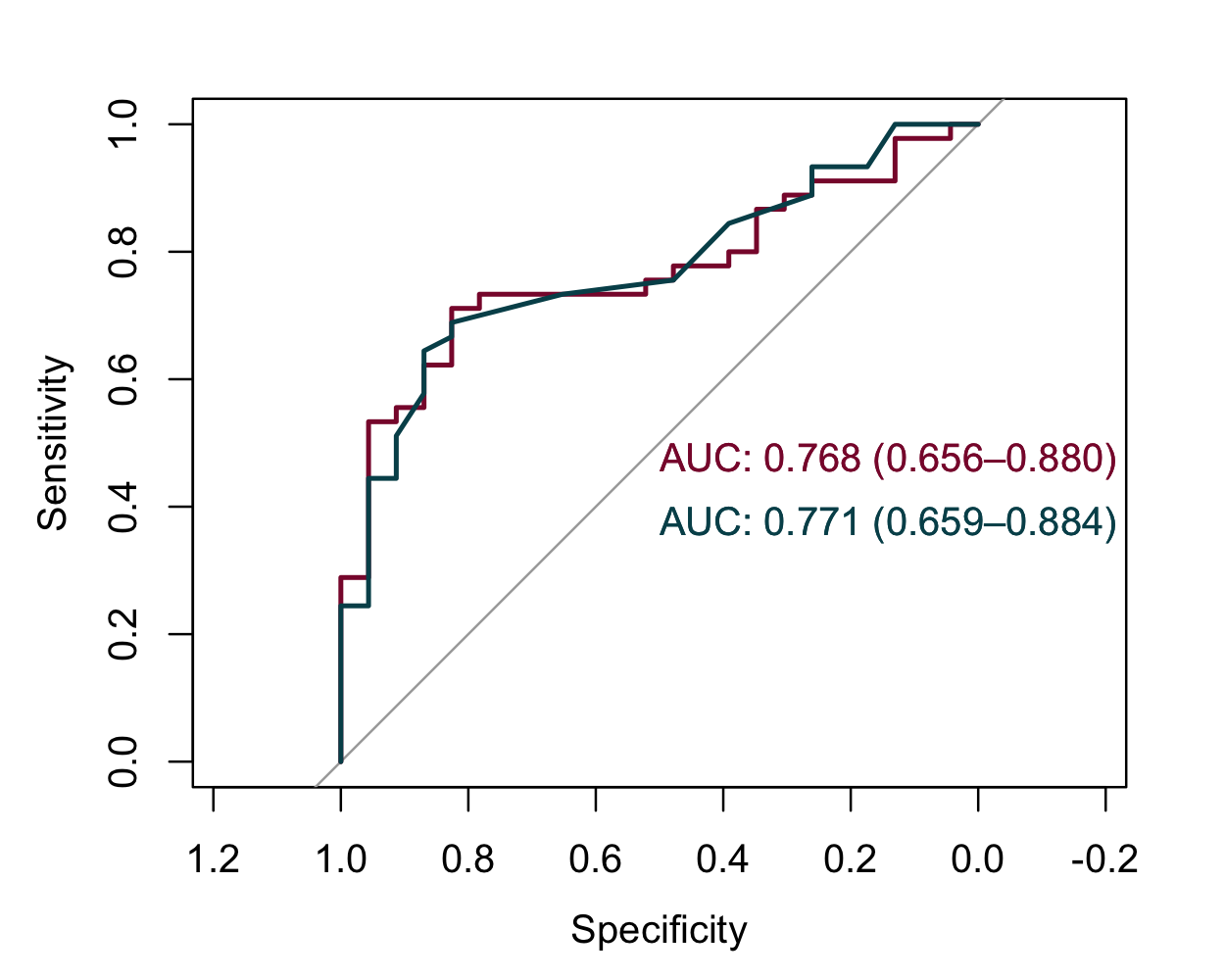


**Supplementary Figure 2.** AUC of a logistic regression model including only lactate level (blue line) or lactate with phosphate level (dark red line) to detect any seizure. AUC values with 95% confidence intervals are depicted on the graph. **Abbreviations:** AUC, area under the curve

**Supplementary Table S1.** Absolute low phosphate levels detected in seizure types with or without a convulsive component. Fisher’s exact test to compare the two groups was non-significant (p-value = 0.07).

|  | **Low Phosphate < 0.8 mmol/L** | |
| --- | --- | --- |
| **Seizure type** | **No** | **Yes** |
| No convulsive component^a^ | 16/18 (89%) | 2/18 (11%) |
| Convulsive component^b^ | 27/42 (64%) | 15/42 (36%) |
| ^a^includes: focal, focal aware, focal unaware, generalised without convulsion  ^b^includes: generalised convulsive seizure, focal to bilateral tonic-clonic, convulsive status epilepticus | | |

**Supplementary Information**

**Stepwise logistic regression model-lactate, calcium and phosphate as predictors of convulsive seizure**

step.model_audit_convulsive <- multivariate_audit_convulsive %>% stepAIC(trace = FALSE)

summary (step.model_audit_convulsive)

Call:

glm(formula = Seizure_yes_no ~ Ca_mmol.L + Phos_mmol.L + Lactate_mmol.L,

family = binomial, data = audit_convulsive)

Coefficients:

Estimate Std. Error z value Pr(>|z|)

(Intercept) 12.0277 8.4688 1.420 0.1555

Ca_mmol.L -4.7437 3.4148 -1.389 0.1648

Phos_mmol.L -2.2294 1.3859 -1.609 0.1077

Lactate_mmol.L 0.9907 0.3946 2.511 0.0121 *

---

Signif. codes:

0 ‘***’ 0.001 ‘**’ 0.01 ‘*’ 0.05 ‘.’ 0.1 ‘ ’ 1

(Dispersion parameter for binomial family taken to be 1)

Null deviance: 77.904 on 57 degrees of freedom

Residual deviance: 55.109 on 54 degrees of freedom

AIC: 63.109

Number of Fisher Scoring iterations: 6

exp(coef(step.model_audit_convulsive))

(Intercept) Ca_mmol.L Phos_mmol.L Lactate_mmol.L

1.673202e+05 8.706764e-03 1.075883e-01 2.693195e+00

1-pchisq (77.904-55.109, 57-54)

[1] 4.45575e-05

ors_multivariate_convulsive<- exp(coef(step.model_audit_convulsive))[2:4]

cis_multivariate_convulsive <- exp(confint.default(step.model_audit_convulsive))[2:4,]

cbind(ors_multivariate_convulsive, cis_multivariate_convulsive)

ors_multivariate_convulsive 2.5 %

Ca_mmol.L 0.008706764 1.079405e-05

Phos_mmol.L 0.107588286 7.114142e-03

Lactate_mmol.L 2.693194927 1.242672e+00

97.5 %

Ca_mmol.L 7.023105

Phos_mmol.L 1.627075

Lactate_mmol.L 5.836855

**Logistic regression model – absolute low phosphate alone as predictor**

Call:

glm(formula = Seizure_yes_no ~ Phos_low, family = binomial, data = audit_all_phos)

Coefficients:

Estimate Std. Error z value Pr(>|z|)

(Intercept) 0.4290 0.2428 1.767 0.0773 .

Phos_lowYES 1.7111 0.7860 2.177 0.0295 *

---

Signif. codes:

0 ‘***’ 0.001 ‘**’ 0.01 ‘*’ 0.05 ‘.’ 0.1 ‘ ’ 1

(Dispersion parameter for binomial family taken to be 1)

Null deviance: 114.57 on 89 degrees of freedom

Residual deviance: 108.02 on 88 degrees of freedom

AIC: 112.02

Number of Fisher Scoring iterations: 4

exp(coef(univariate_low_phos2))

(Intercept) Phos_lowYES

1.535714 5.534884

1-pchisq (114.57-108.2, 89-88)

[1] 0.01160656

ors_uni_low_phos2 <- exp(coef(univariate_low_phos2))

cis_uni_low_phos2 <- exp(confint.default(univariate_low_phos2))

cbind(ors_uni_low_phos2, cis_uni_low_phos2)

ors_uni_low_phos2 2.5 % 97.5 %

(Intercept) 1.535714 0.9541281 2.471805

Phos_lowYES 5.534884 1.1860248 25.829930
